# Impact of Cluster Zone Leaf Removal on Grapes cv. Regent Polyphenol Content by the UPLC-PDA/MS Method

**DOI:** 10.3390/molecules21121688

**Published:** 2016-12-11

**Authors:** Kamila Mijowska, Ireneusz Ochmian, Jan Oszmiański

**Affiliations:** 1 Department of Horticulture, West Pomeranian University of Technology Szczecin, Słowackiego 17 Street, 71-434 Szczecin, Poland; ireneusz.ochmian@zut.edu.pl; 2 Department of Fruit and Vegetable Processing, Wroclaw University of Environmental and Life Sciences, Chełmońskiego 37 Street, 51-630 Wrocław, Poland; oszm@wnoz.up.wroc.pl

**Keywords:** grapevine, defoliation, UPLC-PDA/MS, anthocyanins, flavan-3-ols, flavonols, phenolic acids, fruit quality

## Abstract

Leaf removal is known to enhance light exposure of clusters and therefore may affect grape composition. Owing to the risk of decreasing grape quality or sunburn as a consequence of improper sun exposure, it is crucial to determine the optimum leaf removal techniques adequate for the particular climate conditions of a vineyard area. Defoliation experiments on vine cv. Regent were conducted in two consecutive years (2014 and 2015). The effect of leaf removal treatment on the qualitative and quantitative composition of the polyphenol compounds in the grapes, with reference to the basic weather conditions of the vineyard area, located in Szczecin in the North-Western part of Poland, was assessed. Defoliation was performed manually in the cluster zone at three phenological plant stages: pre-flowering, berry-set and véraison. Leaf removal, especially early defoliation (pre-flowering), enhanced total polyphenol content, including the amount of anthocyanins, flavonols and flavan-3-ols and furthermore, it increased the amount of soluble solids, decreasing the titratable acidity in grapes. On the other hand, the treatments had a reducing impact on the phenolic acids in berries. Defoliation at earlier stages of cluster development appears to be an efficient strategy for improving berry quality in cool climate areas, however, additionally further weather data control is required to determine the effects on berry components.

## 1. Introduction

Grapes (*Vitis vinifera* L.) are one of the largest fruit crops and most commonly consumed fruits in the world, belonging to a group of foods rich in antioxidant compounds [1]. Furthermore, an increasing number of human clinical trials has demonstrated that the consumption of grapes and their products, such as grape juice or wine, exerts many health-promoting effects particularly the reduced risk of cardiovascular diseases, type-2 diabetes, and other chronic complications [1]. These effects result from the fact that, grapes contain many active phenolic compounds that possess antioxidant, antimutagenic [2,3], cardioprotective, anticancer, antiaging, antimicrobial [4] and anti-inflammatory properties [4,5]. According to these properties, the most important grape polyphenols are anthocyanins, flavanols (also called flavan-3-ols), flavonols [6] and phenolic acids [7]. Due to their biological and organoleptic characteristics, berry phenolics have a significant impact on wine quality and such grape extracts are used as sources of natural compounds in the pharmaceutical, food and nutraceutical industries [8]. The composition of phenolic compounds in berries depends on grape varieties, cultural [8] and agronomical practices [9,10] as well as climate [11]. Grape maturity index is also a very important parameter because quantitative and qualitative modification of anthocyanins and tannins occurs during ripening [12,13]. The suitable ripeness and health of grapes are closely linked to vine yield, cluster exposure and foliage density [14]. Leaf removal, widely used in cool and wet climates, is known to improve the light exposure of clusters, enhancing air circulation and berry temperature [15,16,17]. Application of this treatment before flowering has been shown to be useful in enhancing grape composition and wine quality [17]. In Mediterranean environments, leaf removal is usually performed in July during the break-out of colour [18]. Besides improving grape quality, leaf removal could have a role in attenuating vine diseases [15,16,18]. Inadequate sunlight exposure can result in poor quality grapes on the other hand over-exposure to sunlight leads to fruit sunburn and inhibition of colour development [15]. Additionally, temperature affects the rate of development and the loss of various biochemical compounds in grapes, including the accumulation of sugar, the loss of acids through respiration and the synthesis and maintenance of colour and flavour compounds [19]. Thus, to achieve high quality crops it is important to determine optimum leaf removal techniques and also take into consideration the critical influence of climate conditions [15].

The aim of this paper is to evaluate the influence of leaf removal treatment on the qualitative and quantitative composition of polyphenol compounds in cv. Regent grapes, with reference to the basic weather conditions of a vineyard area located in Szczecin, in the North-Western part of Poland.

## 2. Results and Discussion

### 2.1. Vineyard Weather Data and Treatment Time

Considerable differences in the weather of the particular years (2014 and 2015) and significant deviations of these two years values from the average growing season during the multi-year period (1951–2012) were observed. These changes are summarized in Table 1. 

The average growing season temperature between 1951 and 2012 (13.7 °C) was exceeded by 1.2 °C and 1.5 °C, respectively, in the experimental years 2015 and 2014. Schernewski [21] used the Szczecin Lagoon as an example to demonstrate that between 1950 and 1989, the average growing season temperature was 13 °C. During nine years in this period the temperature did not even reach 12.5 °C. From 1990 to 2006 the average growing season temperature was 13.8 °C and during the last 10 years of this period, the temperature never fell below 13.5 °C. Assuming an average increase of temperatures during the next 50 years of 2 °C during the growing season, the entire Danish, German, Polish, Latvian and Lithuanian Baltic Sea coast could provide suitable conditions for viniculture. The boundaries for viniculture might expand to a northern latitude of 60° [21].

Generally, the basic weather parameters such as average temperatures, sun hours and cloudiness were at a similar level during the 2014–2015 growing seasons. Exceptionally little precipitation was observed during the 2015 growing season (242 mm), compared to the years 1951–2012 and the 2014 growing season (391 and 448 mm respectively). Specifically, the period from April to July (growth of green mass, flowering initiation and berry growth) in 2015 was characterised by lower average temperatures, less sun hours and more cloudiness relative to the same period in 2014. These conditions could have limited the vine development, and delay flowering stage. Thereupon, in 2015 the PF stage took place a month later compared to 2014. The biggest deviation in the weather of 2015 took place in August, when rainfall was extremely low (14.7 mm) compared to August in the average growing season during the multi-year period and 2014 (74.2 and 104.6 mm respectively). Additionally in August 2015, when grapes achieved the VE stage, the most sun hours (278 h) were noted, the least cloudiness (17%) as well as the highest number of days with a temperature over 30 °C (11 days). All these parameters can have an effect on the development of vines and their particular phenological stages, with consequences on berry quality. 

### 2.2. Physico-Chemical Attributes of Berries

Maturity indices such as TSS and TA as well as cluster weights and the weights of 100 berries are shown in Table 2. Berries collected in 2015 were characterised by higher TSS and TA contents (21.8 °Brix, 8.48 g/L) relative to 2014 (19.9 °Brix, 7.41 g/L). In 2014, the concentration of soluble solids was statistically insignificant with the exception of grapes under PF treatment which were more abundant in TSS. In 2015, berries from vines defoliated at VE had statistically lower levels of soluble solids. Grapes with less exposure to sunlight had a significantly higher content of TA in the both examined years.

Baiano et al. [22] found grapes of defoliated vines to generally have higher sugar content and lower acidity. This is in line with our results indicating that grapes under PF treatment (early defoliation) showed the highest content of TSS (21.2 °Brix) and the lowest content of TA (7.80 g/L). Berries under VE treatment achieved the lowest TSS level (20.5 °Brix), and the highest TA level (8.03 g/L) compared to the control sample (8.10 g/L). As reported by Intrigliolo et al. [23] for southeastern Spain (cv. Mandó), total soluble solids concentration showed a tendency to increase in defoliated vines, while titratable acidity and pH were not clearly affected by leaf pulling applied procedures. In contrast, Kotseridis et al. [24] observed that fruit zone leaf removal did not affect must soluble solids. 

In general terms berry and cluster weights as well as pH tended to remain unaffected by defoliation. These parameters varied more between seasons and were statistically higher for berry weight (196 g) and pH (3.80) in 2014, and for cluster weight (139 g) in 2015. Statistical differences regarding defoliation in the case of berry and cluster weight were observed in the particular growing seasons but were ambiguous and it was not possible to make inferences from the data. Our results are consistent with the observation of Intrigliolo et al. [23], who noted more of an effect of seasons on berry weight than of defoliation. In contrast, Palliotti et al. [17] observed smaller clusters and berries as well as fewer berries per cluster after defoliation. As reported by Kotseridis et al. [24], depending on the cultivar, defoliation might have a different impact, if at all, on cluster weight.

### 2.3. Polyphenol Compounds

**Figure 1:**
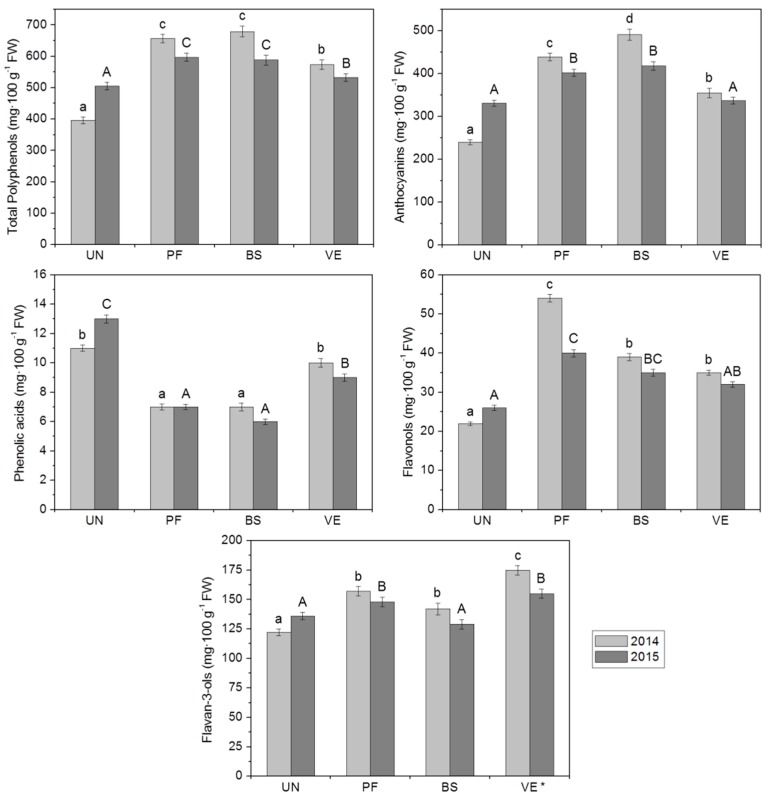
The polyphenol content in mg·100 g^−1^ FW of grape cv. Regent in the two years studied, as related to different leaf removal treatments. * UN: Untreated (no leaf removal), PF: Pre-flowering, BS: Berry-set, VE: Véraison. Data represented by mean ± SD: standard deviation. Means having same letter were not significantly different by Tukey’s comparison *p* < 0.05 level. Lowercase letters indicate the means of 2014, capital letters of 2015.

Differences in grape polyphenol content were noted between growing seasons (Figure 1). In 2015, as compared to 2014, grapes from defoliated vines had a lower total polyphenol content as well as a lower content for each subgroup (anthocyanins, phenolic acids, flavonols and flavan-3-ols). In contrast, berries from UN plants (shaded conditions) reached a higher polyphenol content in 2015, including each subgroup, than in the 2014 growing season. Generally, total polyphenol content as well as the amount of anthocyanins, flavonols and flavan-3-ols in grapes under defoliation were statistically higher than in the control samples, in both years studied. An exception occurred in 2015, when the contents of anthocyanins and flavonols in berries under VE treatment and the content of flavan-3-ols in berries under BS treatment were statistically similar compared with controls. Grapes from non-defoliated vines showed the highest phenolic acids levels in both years. Greater differences between the content of total polyphenol and each subgroup in all fruit samples were seen in 2014. There were no differences in the qualitative composition of berries’ polyphenol compounds between the growing seasons, or between treatments. Identification of 33 compounds belonging to the anthocyanins, phenolic acids, flavonols and flavan-3-ols was based on a comparison of their retention times, MS and MS/MS data, with available standards and published data [25,26]. The results are presented in Table 3. 

The polyphenol content of berries significantly changed under treatment compared to those which were not affected by defoliation. The results are presented in Table 4. The content of polyphenol compounds in fruits varied according to leaf removal times. Total polyphenol content in grapes was noted as between 450.51 to 633.55 mg·100 g^−1^ FW, respectively, for controls and BS leaf removal. BS and PF treatments occurred in the same statistical group and showed approximately a 40% higher total polyphenol content in berries compared to controls, and in the case of VE treatment an increase of 23% was noted. 

Substantial differences in the percentage of particular subgroup compounds were observed. In the total polyphenol content, anthocyanins accounted for 62% to 72%, flavan-3-ols from 21% to 30%, flavonolos from 5% to 7% and phenolic acid from 1% to 3%. According to these findings, the highest percentage of anthocyanins as well as the lowest percentage of phenolic acids and flavan-3-ols occurred in the case of BS treatment. Additionally, the highest percentage of phenolic acids, but the lowest percentage of flavonols were found in UN berries. Fruits that underwent PF treatment were found to have the highest percentage of flavonols. On the other hand, berries which underwent VE defoliation had the highest percentage of flavan-3-ols.

#### 2.3.1. Anthocyanins

Environmental effects have a greater impact on the anthocyanin content in grapes than on their composition, which is more closely linked to the vine cultivar [3]. As reported by Yamane et al. [27], anthocyanin accumulation in grape skins was significantly higher at 20 °C than at 30 °C and the most sensitive stage for temperature was from one to three weeks after colouring began. This is in line with our outcomes, that in 2015 grapes under treatments were less abundant in anthocyanins as compared to those in 2014 (Figure 1). As mentioned in Section 2.1, the period of berry ripening in 2015 was characterised by the most sun hours and the highest number of days with temperatures over 30 °C (11 days). According to Mori et al. [28], a decrease of anthocyanins in grape skins under high temperatures (35 °C) could be caused by many factors, such as chemical and/or enzymatic degradation, as well as inhibition of anthocyanin biosynthesis. In other experiments, Goto-Yamamoto et al. [29] reported that a high temperature (35 °C in the daytime/25 °C at night time) after véraison significantly reduced anthocyanin concentration in berry skins and modified their composition, as well as moderately reduced proanthocyanidin and quercetin concentrations.

In the grapes studied, fifteen anthocyanin compounds were identified. Their content ranged from 285.28 to 454.56 mg·100 g^−1^ FW (Table 4), respectively for controls and BS treatment. The anthocyanin content in grapes under PF leaf removal was statistically similar to BS treatment (420.25 mg·100 g^−1^ FW). The anthocyanin profile of grapes was affected by leaf removal timing. In comparison to controls, fruits under defoliation achieved a 20% to 60% higher anthocyanin content, respectively, for VE and BS treatments. Sternad Lemut et al. [30] noted that in both years studied, anthocyanin content in wines was higher as compared with controls (in 2009 wines it was 18% and 11% higher in the case of véraison and berry-set treatments respectively; in 2010 wines it was 50% and 43% higher in the case of berry-set and pre-flowering treatments respectively). In other studies on grapes of the ‘Regent’ variety cultivated in the wine-growing region of Moravia (EU wine-growing zone B), where the average annual temperature is 9.42 °C and the average precipitation is 510 mm, Balík et al. [31] found the content of anthocyanins to be 1.81 g·kg^−1^.

In our study, defoliation allowed achievement of berries with a higher concentration of cyanidin, malvidin, peonidin and petunidin derivatives, with the exception of delphinidin derivatives, which were higher in control samples. Generally, the anthocyanin compounds found most frequently in the ‘Regent’ fruits were the 3-*O*-glucoside forms of delphinidin, petunidin, malvidin, cyanidin and peonidin, in this order. Looking at the profiles of these compounds, the highest value for each was under BS treatment, followed by PF, VE and UN. In the case of cyanidin-3-*O*-glucoside and petunidin-3-*O*-glucoside, PF treatment showed statistically similar results compared to BS. Control was characterised by the lowest content of each 3-*O*-glucoside form. In the case of cyanidin-3-*O*-glucoside and malvidin-3-*O*-glucoside, there were no statistical significant differences between berries at VE and UN. According to the results of Sternad Lemut et al. [32] at the Potoce vineyard (Slovenia), the highest concentration of 3-*O*-glucoside forms in berry skins was in the case of berry set. 

Looking at our results, the 3-*O*-acethyl-glucoside and 3-*O*-(6-*p*-coumaroyl)-glucoside forms of delphinidin were the highest in control, and exceeded approximately four and five times respectively the concentration of these compounds in berries under leaf removal. Additionally, the 3-*O*-acethyl-glucoside forms of cyanidin and petunidin showed a statistically relevant decrease in their amount in berries from defoliated vines. The other 3-*O*-acethyl-glucoside and 3-*O*-(6-*p*-coumaroyl)-glucoside forms were increased under leaf removal, especially at PF treatment.

#### 2.3.2. Phenolic Acids

Seven different compounds belonging to the group of phenolic acids were identified. Defoliation significantly decreased phenolic acids in cv. Regent berries, especially by reducing of *trans*-caftaric acid and gallic acid. The content of phenolic acids that was observed in controls (11.98 mg·100 g^−1^ FW), exceeded the amounts of these compounds in grapes under BS and PF treatments by nearly two times. In the case of VE treatment, the smallest decrease of these compounds relative to the control sample was noted. In a study on cv. Nebbiolo grapes, Nicoletti et al. [33] observed that the concentration of phenolic acids (caftaric and coutaric acids) on a dry mass basis in grapes was significantly higher in berries from vines that were defoliated post-véraison, followed by those not defoliated and those defoliated at fruitset. As reported by Bubola et al. [34], the concentration of total hydroxycinnamic acids was the highest in samples treated at berry set stage, but it did not differ significantly among control, berry set and veraison samples, while before blooming stage treatment resulted in significantly lower concentrations of total hydroxycinnamic acids. In turn, Sternad Lemut et al. [32] noted that content of hydroxycinnamic acids (*trans*-caftaric, *cis*- and *trans*-coutaric acids) in grapes changed only slightly under leaf removal. Furthermore authors observed that defoliation may result in different phenolic accumulation trends related to many factors, such as site, row orientation, exposition and canopy architecture.

Similarly, as reported by Ehrhardt et al. [35], in our study *trans*-caftaric acid was one of the most abundant of the phenolic acids, especially occuring in the UN samples (5.25 mg·100 g^−1^ FW). Additionally, in the case of controls, statistically higher levels of *cis*-fertaric and gallic acids were found as well. 

#### 2.3.3. Flavonols

Among the flavonols, derivatives of quercetin, myricetin, isorhamnetin and kaempferol were found in the fruits. Six compounds were identified. The quantity of flavonols in ripe berries is mainly influenced by environmental parameters, as a consequence of their photo-protective effect against excessive direct sunlight [3]. Biosynthesis of flavonols was observed during the development of the berries, with the biggest increase recorded 3–4 weeks after the fruit started to ripen, i.e., during the post-véraison period [36]. In our study, defoliation enabled an increase in flavonol content relative to UN. Berries from vines under PF treatment were characterised by the highest content of flavonols (46.90 mg·100 g^−1^ FW); and amount nearly two times higher than for controls (23.98 mg·100 g^−1^ FW). In Baiano et al.’s [22] study, the flavonols increased as a consequence of all defoliation treatments at full véraison. Sternad Lemut et al. [30] noted that total flavonols in 2009 wines were 71% and 52% higher in the case of véraison and berry-set treatments respectively, as compared with untreated controls, while in 2010 the average content of flavonols was higher with pre-flowering leaf removal (75% higher than controls). As reported by Spayd et al. [37], sun-exposed grapes *V. vinifera* ‘Merlot’, contained almost a ten times greater concentration of total flavonols than shaded clusters. Moreover, exposure to solar radiation increased the content of the 3-*O*-glycosides of quercetin, kaempferol, and myricetin [37]. 

In our study, the most marked changes in compound amounts under defoliation was observed in the case of myricetin-3-*O*-glucoside, quercetin-3-*O*-glucoside, quercetin-3-*O*-glucuronide and isorhamnetin-3-*O*-glucoside. All of the identified flavonol compounds achieved the highest content under PF treatment followed by BS and VE, with the exception of myricetin-3-*O*-glucoside, which was the highest in the order BS > VE > PF. Leaf removal at VE did not have any effect on the concentration of quercetin-3-*O*-rutinoside and kaempferol-3-*O*-glucoside compared to control.

#### 2.3.4. Flavan-3-ols

Flavan-3-ols, the second largest subgroup after anthocyanins, involved five identified compounds: B-type procyanidins, (+)-catechin and (−)-epicatechin. Defoliation significantly increased the content of flavan-3-ols in berries under PF and VE treatments relative to control (152.68, 164.86 and 129.27 mg·100 g^−1^ FW, respectively). Under BS leaf removal, an increase in these compounds was noted as well, but it was not statistically significant (135.70 mg·100 g^−1^ FW). 

Nevertheless, clusters with more sun exposure were especially characterised by an increase in catechin. Berries under PF treatment revealed the highest concentration of catechin (77.42 mg·100 g^−1^ FW), nearly twice as high as UN control (39.17 mg·100 g^−1^ FW). In the case of epicatechin and procyanidin, B2 cluster zone leaf removal led to a decrease in their amounts, as compared to shaded grapes. UN control were the most abundant in terms of epicatechin and procyanidin B2—42.40 and 31.19 mg·100 g^−1^ FW, respectively. With respect to other B-type procyanidins, there was no clear correlation to light conditions. 

In Kotseridis et al.’s [24] paper, on the different severities of leaf removal in the fruit zone of *V. vinifera* cultivars, defoliation significantly reduced seed flavan-3-ols (mainly catechin and epicatechin) as compared with nondefoliated samples. However, as reported by Feng et al. [15], there were no differences observed for the flavan-3-ols, including catechin and epicatechin, between different intensities of leaf removal treatments.

## 3. Materials and Methods

### 3.1. Characteristics of the Research Area and Experimental Design

The investigation was carried out in two consecutive years (2014 and 2015) at a research station of the West Pomeranian University of Technology in Szczecin. The station is located in the North-Western part of Poland in the Szczecin Lowland at a distance of approximately 65 km from the Baltic Sea (53°40′ N, 14°46′ E, EU wine-growing zone A).

The study involved a dark-skinned grapevine (cv. Regent), which is a German interspecific hybrid well-known and recommended for cool climate areas and, as reported by Lisek [38], one of the most reliable cultivars for commercial wine production in Poland. The vines were grafted on SO_4_ rootstock and planted in 2010 with a North-South row orientation at 2.3 m × 1 m. The plants were pruned with a Guyot (one arm) training system and vertically positioned with eight shoots with two clusters per each. Other standard vineyard management practices, including pest treatment, were performed during both growing seasons.

The experimental treatments were arranged in a randomised complete 4-block design. Each experimental unit was comprised of 10 vines. Defoliation was performed manually in the cluster zone, removing the basal four to six leaves from all shoots. This resulted in approximately 30–40 centimetres of free space without leaves. Treatment at three phenological plant stages was carried out according to the method of Sternad Lemut et al. [30]: pre-flowering (PF), berry-set (BS) and véraison (VE). One-fourth of the experimental vines were left untreated (UN) and used as a control. Pre-flowering treatment was performed on 11 June and 1 July, berry-set treatment on 10 and 9 July, and véraison on 22 and 30 August in 2014 and 2015 respectively. All leaf removal was maintained until harvest. Fruits were collected at physiological maturity on 6^th^ and 12^th^ of October in successive seasons.

### 3.2. General Grape Parameters

The weights of clusters and berries were measured with WPX 4500 (RADWAG, Radom, Poland) electronic scales (0.1 g accuracy). Total soluble solid (TSS) content of grapes was determined as °Brix, using a PAL-1 (Atago, Tokyo, Japan) refractometer. The pH of the grape juice samples was measured using a pH-meter (Elmetron 501, Zabrze, Poland). Titratable acidity (TA) was determined by titration of a water extract of juice with 0.1 N NaOH to an end point of pH 8.1. To calculate cluster weight, 30 clusters per treatment and control were randomly chosen. The same clusters were used to calculate other parameters. The measurements of berries weight, TSS, pH and TA were performed on three replicates of 100 fresh berries randomly chosen. All grape parameters were measured immediately after harvest.

### 3.3. Polyphenol Content

#### 3.3.1. Reagents and Standards

Formic acid and methanol were purchased from Sigma-Aldrich (Steinheim, Germany). Acetonitrile was purchased from Merck (Darmstadt, Germany). Quercetin-3-*O*-glucoside, quercetin-3-*O*-rutinoside, kaempferol-3-*O*-glucoside, myricetin-3-*O*-glucoside, isorhamnetin-3-*O*-glucoside, cyanidin-3-*O*-glucoside, peonidin-3-*O*-glucoside, (−)-epicatechin, (+)-catechin, procyanidins, gallic acid were purchased from Extrasynthese (Lyon, France).

#### 3.3.2. Extraction Procedure

Three replicates of 20 randomly chosen berries were kept frozen at −27 °C until analysis, then prepared according to the methodology of Oszmiański et al. [39]. The fruits were extracted with methanol acidified with 2.0% formic acid. The separation was conducted twice by incubation for 20 min under sonication (Sonic 6D, Polsonic, Warsaw, Poland) followed by shaking from time to time (a few times or rarely). Subsequently, the suspension was centrifuged MPW-251 (MPW MED. INSTRUMENTS, Warsaw, Poland) at 19,000× *g* for 10 min. Prior to analysis, the supernatant was additionally purified with a Hydrophilic PTFE 0.20 µm membrane (Millex Samplicity Filter, Merck).The polyphenol content in each extract was specified by means of the ultra-performance liquid chromatographyphoto-diode array detector-mass spectrometry (LC-PDA-MS, Waters Corporation, Milford, MA USA) method. All extractions were carried out in triplicate.

#### 3.3.3. Identification of Phenolic Compounds with the UPLC-PDA/MS Method

Analyses were performed according to the methodology of Oszmiański et al. [39]. In ‘Regent’ grape extracts’, polyphenol identification was executed by using an ACQUITY Ultra Performance LC system appointed with a binary solvent manager, a photodiode array detector (Waters Corporation, Milford, MA, USA) and a G2 Q-TOF micro mass spectrometer (Waters, Manchester, UK) equipped with an electrospray ionisation (ESI) source operating in both negative and positive modes. Individual polyphenol separations were executed using a UPLC BEH C18 column (1.7 µm, 2.1 mm × 100 mm, Waters Corporation, Milford, MA) at 30 °C. The elution of injected samples (10 µL) was carried out in 15 min, followed by a sequence of linear gradients and isocratic flow rates of 0.45 mL·min^−1^. The mobile phase consisted of solvent A (0.1% formic acid, *v*/*v*) and solvent B (100% acetonitrile). The examination commenced with an initial isocratic elution of 99% solvent A (0–1 min), while applying a linear gradient for 12 min lowering solvent A to 0%. Next, at 12.5 to 13.5 min, the gradient was returned back to the initial composition (99% A), with the tore-equilibrate column being kept constant. The overall analysis was based on full data-dependant MS scanning with a *m/z* range from 100 to 2500. The reference compound used for the examination was Leucine encephalin, at a concentration of 500 pg/µL and a flow rate of 2 µL/min. The [M − H]^−^ ion at 554.2615 Da and [M + H]^+^ ion at 556.2771 were detected. The [M − H]^−^/[M + H]^+^ ions were recognised during a 15 min analysis performed within ESI-MS accurate mass experiments, which were constantly introduced via the LockSpray channel using a Hamilton pump. The mass spectrometer operated in both negative-and positive-ion modes, adjusted to base peak intensity (BPI) chromatograms, scaled to 12,400 counts per second (cps) (100%) within a locked mass correction of ±1.000 for the mass window. The MS conditions were optimised according to the following parameters: a capillary voltage of 2500 V, a cone voltage of 30 V, a source temperature of 100 °C, a desolvation temperature of 300 °C and a desolvation gas (nitrogen) flow rate of 300 L/h. Collision-induced fragmentation experiments were performed using argon as the collision gas, with voltage ramping cycles from 0.3 to 2 V. Characterisation of each and every single component was conducted via retention time and accurate molecular masses while individual compounds were optimised to their estimated molecular mass in both negative and positive modes, prior to and as a result of fragmentation. Afterwards, the data collected from UPLC-MS were uploaded to the MassLynx 4.1 ChromaLynx Application Manager software (MassLynx 4.1 SCN802, Waters Corporation, Milford, MA USA). Quantification was achieved by injection of solutions of known concentrations ranging from 0.05 to 5 mg/mL (R^2^ ≤ 0.9998) of phenolic compounds as standards. The results were expressed as mg per 100 mL for must.

Based on the data delivered, the software itself has been developed to scan multiple samples for the defined substances. The various data analysis runs were monitored at the following wavelengths: flavan-3-ols at 280 nm, phenolic acids at 320 nm, flavonol glycosides at 360 nm and anthocyanins at 520 nm.

The PDA spectra were measured over a wavelength range of 200–600 nm in steps of 2 nm. Finally, the retention times and spectra were compared with authentic standards.

### 3.4. Statistical Analysis

All statistical analyses were performed with Statistica 12.5 (StatSoft Polska, Cracow, Poland). The data were subjected to one and two-factor variance analysis (ANOVA). Mean comparisons were performed using Tukey’s least significant difference (LSD) test; significance was set at *p* < 0.05.

## 4. Conclusions

Leaf removal had a significant influence on grape cv. Regent parameters in the weather condition of Szczecin, Poland. Defoliation led to higher concentrations of soluble solids and lower amounts of titratable acidity in grapes, especially in the case of early treatment at pre-flowering. However, defoliation did not have a significant influence on berry and cluster weights or on pH.

Generally, defoliation increased the total polyphenol content as well as the amount of anthocyanins, flavonols and flavan-3-ols in grapes. On the other hand, leaf removal decreased the content of phenolic acids, especially the amounts of *trans*-caftaric, *cis*-fertaric acids and gallic acid. The quantitative composition of polyphenol compounds in fruits was affected by leaf removal timing. In the case of pre-flowering and berry-set treatments, the highest values of total polyphenol compounds and anthocyanin content was observed. Additionally, defoliation at the pre-flowering stage led to the greatest increase in flavonols, and next to véraison treatment, showed the highest increase of flavan-3-ols level in grapes.

To sum up, defoliation at earlier stages of cluster development appears to be an efficient strategy for reaching higher polyphenol content in grapes in cool climate areas, though further control of weather data that has still a great effect on total soluble solids and titratable acidity is required.

## Figures and Tables

**Table 1:** Weather conditions during the vegetative season (April–October) in the years 2014–2015 with reference to the average growing season during the multi-year period (1951–2012) [20].

Year	Month							
	April	May	June	July	August	September	October	
	Average Temperature (°C)							Mean
2014	10.8	13.4	16.3	21.3	17.5	15.4	11.8	15.2
2015	8.7	12.5	15.6	18.6	21.1	14.1	13.7	14.9
1951–2012	8.0	13.0	16.4	18.2	17.6	13.8	9.2	13.7
	**Rainfall (mm)**							**Total**
2014	47.5	85.3	26.5	70.8	104.6	80.9	32.8	448
2015	29.0	48.0	32.8	62.0	14.7	34.4	22.1	242
1951–2012	39.7	62.9	48.2	69.6	74.2	58.7	37.3	391
	**Number of Days with Temperature Over 30 °C**							**Total**
2014	-	-	1	8	-	1	-	10
2015	-	-	-	3	11	-	-	14
	**Sun Hours**							**Total**
2014	210	213	189	224	123	133	99	1191
2015	136	161	159	197	278	126	131	1188
	**Cloudiness (%)**							**Mean**
2014	29	35	32	21	31	32	37	31
2015	34	31	37	31	17	29	36	31

**Table 2:** General parameters of grape cultivar Regent as related to different leaf removal treatments.

Year	Leaf Removal Treatments *				
	UN	PF	BS	VE	Mean
	Cluster Weight (g)				
2014	95 ± 17 a	106 ± 20 ab	87 ± 14 a	127 ± 19 bc	104 A
2015	125 ± 21 bc	133 ± 23 bc	146 ± 18 c	151 ± 24 c	139 B
mean	110 A	120 A	116 A	139 A	
	**Weight of 100 Berries (g)**				
2014	177 ± 9 bcd	200 ± 11 de	193 ± 11 cde	215 ± 13 e	196 B
2015	161 ± 8 ab	148 ± 7 a	165 ± 10 abc	158 ± 8 ab	158 A
mean	169 A	174 A	179 A	186 A	
	**Total Soluble solid (°Brix)**				
2014	19.5 ± 0.24 a	20.4 ± 0.22 b	19.4 ± 0.21 a	20.2 ± 0.17 a	19.9 A
2015	22.3 ±0.27 c	21.9 ± 0.19 c	22.4 ± 0.21 c	20.7 ± 0.25 b	21.8 B
mean	20.9 AB	21.2 B	20.9 AB	20.5 A	
	pH				
2014	3.88 ± 0.07 c	3.82 ± 0.06 bc	3.77 ± 0.09 bc	3.77 ± 0.05 bc	3.80 B
2015	3.24 ± 0.04 a	3.23 ± 0.05 a	3.29 ± 0.04 a	3.23 ± 0.03 a	3.26 A
mean	3.56 A	3.53 A	3.53 A	3.50 A	
	**Titratable acidity (g/L)**				
2014	7.55 ± 0.08 b	7.36 ± 0.08 ab	7.30 ± 0.09 a	7.42 ± 0.07 ab	7.41 A
2015	8.65 ± 0.11 d	8.24 ± 0.09 c	8.40 ± 0.07 c	8.63 ± 0.07 cd	8.48 B
mean	8.10 B	7.80 A	7.85 AB	8.03 B	

* UN: Untreated (no leaf removal), PF: Pre-flowering, BS: Berry-set, VE: Véraison. Means in same letter are not significantly different at *p* < 0.05 according to Tukey test; lowercase letters indicated interaction and the capital letters main factors, ±SD: standard deviation.

**Table 3:** Characterisation of phenolic compounds of vine grape fruits by retention time, λ_max_ and negative and positive ions using ultra-pressure liquid chromatography with photodiode array and mass spectrometry (UPLC-PDA/MS).

Compounds	Rt (min)	λmax (nm)	(M − H)^+^ (*m/z*)	(MS/MS) (*m/z*)
Gallic acid	1.08	276	169	125
*cis*-Caftaric acid	2.25	328	311	179; 149
*trans*-Caftaric acid	2.58	328	311	179; 149
*cis*-Coutaric acid	2.63	310	295	163; 149
Procyanidin B1	2.76	278	577	289
*trans*-Coutaric acid	3.04	310	295	163; 149
Procyanidin B3	3.12	278	577	289
(+)-Catechin	3.19	276	289	245; 205
*cis*-Fertaric acid	3.57	328	325	193
Procyanidin B2	3.98	278	577	289
*trans*-Fertaric acid	4.00	328	325	193
Delphinidin-3-*O*-glucoside	4.15	526	465+	303
(−)-Epicatechin	4.69	276	289	245; 205
Cyanidin-3-*O*-glucoside	4.85	516	449+	287
Petunidin-3-*O*-glucoside	5.34	527	479+	317
Peonidin-3-*O*-glucoside	6.06	516	463+	301
Malvidin-3-*O*-glucoside	6.42	527	493+	331
Myricetin-3-*O*-glucoside	6.51	343	479	317
Delphinidin-3-*O*-acethyl-glucoside	6.90	529	507+	303
Quercetin-3-*O*-rutinoside	7.11	352	609	463; 301
Quercetin-3-*O*-glucoside	7.26	352	463	301
Quercetin-3-*O*-glucuronide	7.37	352	477	301
Cyanidin-3-*O*-acethyl-glucoside	7.70	517	491+	287
Petunidin-3-*O*-acethyl-glucoside	8.03	529	521+	317
Kaempferol-3-*O*-glucoside	8.04	350	447	285
Peonidin-3-*O*-acethyl-glucoside	8.45	517	505+	301
Isorhamnetin-3-*O*-glucoside	8.51	354	477	315
Malvidin-3-*O*-acethyl-glucoside	8.89	529	535+	331
Delphinidin-3-*O*-(6-*p*-coumaroyl)-glucoside	9.09	527	611+	303
Cyanidin-3-*O*-(6-*p*-coumaroyl)-glucoside	9.66	520	595+	287
Petunidin-3-*O*-(6-*p*-coumaroyl)-glucoside	9.89	527	625+	317
Peonidin-3-*O*-(6-*p*-coumaroyl)-glucoside	10.70	525	609+	301
Malvidin-3-*O*-(6-*p*-coumaroyl)-glucoside	10.84	530	639+	331

**Table 4:** The polyphenol content in mg·100 g^−1^ FW of grape cultivar Regent (average for the years 2014–2015) as related to different leaf removal treatments.

Compounds	Leaf Removal Treatments *			
	UN	PF	BS	VE
Cyanidin-3-*O*-glucoside	45.32 ± 2.91 a	78.97 ± 4.57 b	72.99 ± 5.02 b	54.78 ± 3.14 a
Delphinidin-3-*O*-glucoside	65.85 ± 2.06 a	83.82 ± 3.24 bc	93.49 ± 3.11 c	74.49 ± 2.48 ab
Malvidin-3-*O*-glucoside	51.99 ± 1.79 a	75.71 ± 2.35 b	91.05 ± 2.52 c	61.52 ± 1.90 a
Peonidin-3-*O*-glucoside	42.31 ± 1.33 a	68.46 ± 2.15 bc	75.03 ± 1.98 c	61.18 ± 1.76 b
Petunidin-3-*O*-glucoside	47.01 ± 1.87 a	81.52 ± 1.54 c	89.56 ± 1.72 c	64.54 ± 1.90 b
Cyanidin-3-*O*-acethyl-glucoside	2.56 ± 0.19 b	1.01 ± 0.13 a	1.11 ± 0.11 a	0.82 ± 0.10 a
Delphinidin-3-*O*-acethyl-glucoside	4.56 ± 0.18 b	1.16 ± 0.12 a	1.16 ± 0.10 a	1.04 ± 0.13 a
Malvidin-3-*O*-acethyl-glucoside	2.70 ± 0.0.9 a	4.05 ± 0.10 c	3.89 ± 0.09 bc	3.48 ± 0.08 b
Peonidin-3-*O*-acethyl-glucoside	0.22 ± 0.01 a	0.67 ± 0.02 b	0.75 ± 0.02 b	0.59 ± 0.02 b
Petunidin-3-*O*-acethyl-glucoside	3.51 ± 0.21 c	1.10 ± 0.04 b	0.89 ± 0.03 a	1.17 ± 0.03 b
Cyanidin-3-*O*-(6-*p*-coumaroyl)-glucoside	3.09 ± 0.10 a	5.46 ± 0.10 c	5.77 ± 0.11 c	4.90 ± 0.08 b
Delphinidin-3-*O*-(6-*p*-coumaroyl)-glucoside	6.27 ± 0.28 b	1.24 ± 0.09 a	1.49 ± 0.11 a	1.34 ± 0.09 a
Malvidin-3-*O*-(6-*p*-coumaroyl)-glucoside	4.43 ± 0.13 a	6.94 ± 0.18 b	7.06 ±0.17 b	6.67 ± 0.15 b
Peonidin-3-*O*-(6-*p*-coumaroyl)-glucoside	2.38 ± 0.07 a	4.47 ± 0.12 c	4.36 ± 0.10 bc	4.11 ± 0.11 b
Petunidin-3-*O*-(6-*p*-coumaroyl)-glucoside	3.08 ± 0.09 a	5.67 ± 0.08 b	5.96 ± 0.08 b	5.21 ± 0.10 b
Anthocyanins (%)	285.28 A (63)	420.25 C (67)	454.56 C (72)	345.84 B (62)
*cis*-Caftaric acid	1.61 ± 0.07 c	0.45 ± 0.02 a	0.97 ± 0.03 b	1.42 ± 0.03 c
*trans*-Caftaric acid	5.25 ± 0.16 d	1.66 ± 0.07 b	0.94 ± 0.04 a	2.30 ± 0.08 c
*cis*-Coutaric acid	1.07 ± 0.05 a	2.21 ± 0.08 b	2.01 ± 0.13 b	2.46 ± 0.10 b
*trans*-Coutaric acid	0.51 ± 0.02 a	0.55 ± 0.01 a	0.61 ± 0.02 a	0.50 ± 0.02 a
*cis*-Fertaric acid	0.97 ± 0.05 c	0.34 ± 0.02 a	0.25 ± 0.01 a	0.73 ± 0.03 b
*trans*-Fertaric acid	0.39 ± 0.02 a	0.55 ± 0.01 b	0.54 ± 0.03 b	0.45 ± 0.02 ab
Gallic acid	2.19 ± 0.09 b	1.18 ± 0.06 a	1.03 ± 0.04 a	1.26 ± 0.05 a
Phenolic acids (%)	11.98 C (3)	6.94 A (1)	6.35 A (1)	9.12 B (2)
Myricetin-3-*O*-glucoside	3.50 ± 0.15 a	4.54 ± 0.21 b	6.23 ± 0.30 c	5.43 ± 0.26 bc
Quercetin-3-*O*-rutinoside	3.39 ± 0.17 ab	5.71 ± 0.19 c	3.73 ± 0.22 b	3.07 ± 0.14 a
Quercetin-3-*O*-glucoside	6.95 ± 0.28 a	15.09 ± 0.55 c	11.79 ± 0.37 b	11.31 ± 0.34 b
Quercetin-3-*O*-glucuronide	8.44 ± 0.29 a	16.74 ± 0.41 c	12.05 ± 0.34 b	11.02 ± 0.28 b
Kaempferol-3-*O*-glucoside	0.45 ± 0.03 ab	0.97 ± 0.04 c	0.56 ± 0.04 b	0.44 ± 0.02 a
Isorhamnetin-3-*O*-glucoside	1.25 ± 0.09 a	3.85 ± 0.12 c	2.58 ± 0.11 b	2.53 ± 0.10 b
Flavonols (%)	23.98A (5)	46.90 C (7)	36.94 B (6)	33.80 B (6)
Procyanidin B1	10.87 ± 0.48 a	13.21 ± 0.53 b	10.70 ± 0.44 a	17.47 ± 0.58 c
Procyanidin B2	31.19 ± 1.44 b	23.76 ± 1.31 a	24.16 ± 1.35 a	26.10 ± 1.48 a
Procyanidin B3	5.64 ± 0.21 b	4.45 ± 0.14 a	5.81 ± 0.17 b	9.15 ± 0.24 c
(+)-Catechin	39.17 ± 1.56 a	77.42 ± 2.93 c	64.83 ± 2.38 b	71.32 ± 3.02 bc
(−)-Epicatechin	42.40 ± 2.11 c	33.85 ± 1.76 a	30.20 ± 1.95 a	40.82 ± 1.48 b
Flavan-3-ols (%)	129.27 A (29)	152.68 B (24)	135.70 A (21)	164.86 B (30)
Total polyphenols	450.51 A	626.77 C	633.55 C	553.62 B

* UN: Untreated (no leaf removal), PF: Pre-flowering, BS: Berry-set, VE: Véraison. Means in the same row followed by the same letter are not significantly different at *p* < 0.05 according to Tukey test; ±SD: standard deviation.
